# Kynureninase knockdown inhibits cisplatin resistance in vivo and in vitro and impacts the prognosis of cervical adenocarcinoma

**DOI:** 10.1186/s13008-023-00098-3

**Published:** 2023-09-24

**Authors:** Jun-wen Zhang, Ya-nan Wang, Mei-ling Zhong, Mei-rong Liang

**Affiliations:** 1 Children’s Hospital, Zhejiang University School of Medicine, Hangzhou, 310000 Zhejiang China; 2 Oncology Department of Jiangxi Maternal and Child Health Care Hospital, No. 318, Bayi Avenue, Nanchang, 330006 Jiangxi China

**Keywords:** Kynureninase, Chemotherapy resistance, Cervical adenocarcinoma

## Abstract

**Background:**

Chemotherapy resistance is a leading cause of treatment failure in cases of cervical adenocarcinoma (ADC), and no effective treatment approach has yet been found. We previously identified the differentially expressed kynureninase (KYNU) mRNA in cervical adenocarcinoma cells (HeLa) and cervical adenocarcinoma cisplatin resistance cells (HeLa/DDP) using gene chips. However, the role and potential mechanism of KYNU in the cisplatin resistance of cervical adenocarcinoma remain unclear.

**Methods:**

We verified the expression of KYNU in the cells and tissues of ADC patients and analyzed its correlation with patient prognosis. A stable HeLa/DDP cell line with KYNU mRNA knockdown was constructed. We then used a CCK8 assay to detect cell survival, a transwell assay to evaluate cell migration and proliferation and flow cytometry to measure apoptosis. The effect of KYNU silence on cisplatin sensitivity was evaluated in an orthotopic model of metastatic ADC. Immunohistochemistry was performed to determine the changes in relevant drug resistance-associated protein expression, aiming to explore the underlying mechanism of KYNU-mediated drug resistance.

**Results:**

KYNU is overexpressed in HeLa/DDP cells and tissues and is associated with the poor prognoses of patients with ADC. After KYNU mRNA knockdown, the invasion, migration, and proliferation of HeLa/DDP cells in the cisplatin environment significantly reduced, while the apoptosis rate of HeLa/DDP cells significantly increased. Meanwhile, KYNU knockdown improved the DDP sensitivity of ADC in vivo. Furthermore, silencing KYNU decreased the expressions of CD34 and the drug-resistance related proteins P-gp, MRP1, and GST-π and increased the level of the proapoptotic regulatory protein Bax.

**Conclusion:**

KYNU deficiency enhanced DDP sensitivity by suppressing cell proliferation, migration, and invasion and promoting apoptosis in DDP-resistant ADC cells in vitro. Furthermore, KYNU knockdown improved the drug sensitivity of ADC in vivo. The results showed that KYNU is involved in the chemotherapy resistance of cervical adenocarcinoma.

## Introduction

Cervical cancer is one of the most common malignancies in women and is a serious public health problem in developing countries [1, 2]. With effective screening, the early detection rate of cervical cancer has greatly increased, and its overall morbidity and mortality have decreased; however, the rising incidence and younger onset of cervical adenocarcinoma (ADC) cannot be ignored [3, 4]. Cisplatin-based concurrent chemoradiotherapy is the standard treatment for locally advanced cervical adenocarcinoma. However, cervical ADC has low sensitivity to radiotherapy. In advanced ADC patients who are resistant to cisplatin-based chemotherapy treatment, there is currently no standardized treatment plan for second-line or subsequent treatments, resulting in a very poor prognosis.

Considering the difficulties of clinical treatment, we previously investigated the differential expression of kynureninase (KYNU) mRNA in cervical adenocarcinoma cells (HeLa/DDP) and cervical adenocarcinoma cisplatin-resistant cells (HeLa/DDP) using gene chips. KYNU is a key enzyme in the metabolic pathway of tryptophan. A large number of studies have shown that KYNU plays an important role in carcinogenesis, progression, and chemoresistance [5–7]. However, the role and potential mechanism of KYNU in cisplatin resistance and the malignant progression of cervical ADC remain unclear. In this study, we explored the role of KYNU in the cisplatin resistance of cervical ADC, aiming to provide a theoretical basis for overcoming this resistance and improving the prognoses of patients with cervical ADC.

## Materials and methods

### Tissue samples

A total of 53 paraffin specimens of biopsy tissue were collected from patients with Stage I b1 to Stage II b ADC who received two cycles of platinum-based neoadjuvant chemotherapy at Jiangxi Maternal and Child Health Carehospital. Based on Response Evaluation Criteria in Solid Tumours 1.1 (RECIST1.1), the 53 patients were divided into a DDP-sensitive group (n = 20) and a DDP-resistant group (n = 33). Immunohistochemistry (PV-9000) was performed to determine the expression of KYNU in the biopsy tissues.

### Cell culture

Human cervical adenocarcinoma HeLa cells were preserved in the central laboratory of Jiangxi Maternal and Child Health Hospital. Human cervical ADC cisplatin-resistant HeLa/DDP cells were successfully constructed by our research group in 2014 and stored in a liquid nitrogen tank. HeLa and HeLa/DDP cells were maintained in a DMEM medium (Gibco, UK) supplemented with 10% v/v fetal bovine serum FBS (Gibco, UK). All cell lines were maintained in 37 °C incubators Femented with 5% CO_2_. The HeLa/DDP cells were resuscitated, and stepwise dosing was performed once the cell density was above 70%; the DDP concentrations were 0.2 μg/mL, 0.5 μg/mL, 0.75 μg/mL, and 1 μg/mL. Once the cell state was stable, cisplatin resistance was maintained with 1 μg/mL DDP.

### Lentiviral shRNA-mediated KYNU mRNA knockdown

The HeLa/DDP cells were transfected with the GV248-KYNU lentivirus, involving KYNU mRNA knockdown and the empty vector lentivirus GV248-NC (GenePharma, Shanghai, China). The lentivirus carries a reporter gene—green fluorescent protein (GFP)—which was used to determine transfection efficiency.

### RT-qPCR

Total RNA was extracted from the HeLa and Hela/DDP cells using TRIzol (Invitrogen) and reverse-transcribed to the first-strand cDNA. Quantitative real-time PCR (qPCR) was performed using a StepOnePlus™ real-time PCR system (Applied Biosystems™). The qPCR conditions were as follows: 95 °C for 30 s; 40 cycles of 95 °C for 5 s and 60 °C for 30 s. Quantification was performed using the comparative Ct (ΔΔCt) method according to the manufacturer’s instructions.

### Western blot

Proteins were extracted from the HeLa and Hela/DDP cells using RIPA buffer and qualified using a BCA kit (Solarbio). The proteins were separated via SDS-PAGE and then moved to PVDF membranes. After being blocked with skimmed milk, the target proteins were incubated with primary antibodies, namely anti-KYNU (Abcam, Cambridge, USA.) and anti-P-gp (Abcam, Cambridge, USA). Secondary antibodies were added for 1 h. Finally, the proteins were examined using a chemiluminescence detection system.

### Transwell assay assay

Matrigel, melted into liquid form, was mixed with a serum-free medium at a ratio of 1:8, and 50 μl of the mixture was taken for each well of a 96-well microplate. The wells were placed in a 5% CO_2_ incubator overnight at 37 °C. Then, 200 μl of the cell suspension (1 × 105/ml) was added to the upper chamber, and 500 μl of the medium containing 10% FBS was added to the lower chamber. After incubation in a 5% CO_2_ incubator for 48 h, the cells in the lower layer of the microporous membrane were counted using an optical microscope (100×). The mean cell counts of 10 randomly selected fields were determined.

### Migration assay

The cells were incubated for 24 h in a 5% CO_2_ incubator. They were then stained with crystal violet and photographed under an optical microscope (100×). The numbers of cells in the lower layer of the microporous membrane were counted for 10 randomly selected fields, and the mean values were calculated.

### Cell counting kit-8 (CCK-8) assay

HeLa and HeLa/DDP cells in the logarithmic growth phase were inoculated into 96-well plates (2 × 10^3^ per well). After 24 h, the cells were treated with various concentrations of DDP. A blank group (without cells and DDP) and a control group (without DDP) were included. All experiments were performed in triplicate. After 72 h of incubation, 100 μl of fresh medium was added to the samples. Then, after 2 h, absorbance was measured at 490 nm.

### In vivo tumor xenograft

Animal studies were performed according to the rules for care and use of experimental animals and were approved by the Animal Care Committees Nanchang University (approval numbers: SYXK(赣)2015–0001). A total of 32 female BAlB/c mice were randomly divided into four groups—GV248-KYNU-cisplatin, GV248-NC-cisplatin, HeLa/DDP-cisplatin, and HeLa/DDP-normal saline (NS)—according to whether cisplatin chemotherapy was to be performed. GV248-KYNU and GV248-NC cells were injected subcutaneously into the right axilla of the mice in the first two groups, respectively, and HeLa/DDP cells were injected into the other mice. Once the tumor xenograft grew to about 100 mm^3^, cisplatin chemotherapy was performed intraperitoneally at a dose of 3 mg/Kg every other day for a total of seven days. The volume of the transplanted tumor and the weight of each mouse were measured every three days until their natural deaths. Then, the mice were dissected to determine whether there was tumor metastasis. The tumor specimens were removed for paraffin sectioning, and the pathological changes of the transplanted tumors were observed using hematoxylin–eosin (HE)staining. Immunohistochemistry was performed to detect the expression levels of KYNU, P-gp, GST-π, MRP1, Bax, Bcl-2, and CD34.

### Statistical methods

Quantitative data were recorded as mean ± standard deviation (SD) values. The differences among the groups were analyzed using the Student t-test. The Kaplan–Meier method was used to calculate the survival rate, and a log-rank test was performed to determine the significance of the differences in the survival distribution. *P* value less than 0.05 was defined as statistically significant.

## Results

### KYNU was overexpressed in the HeLa/DDP cells and tissues

**Fig. 1:**
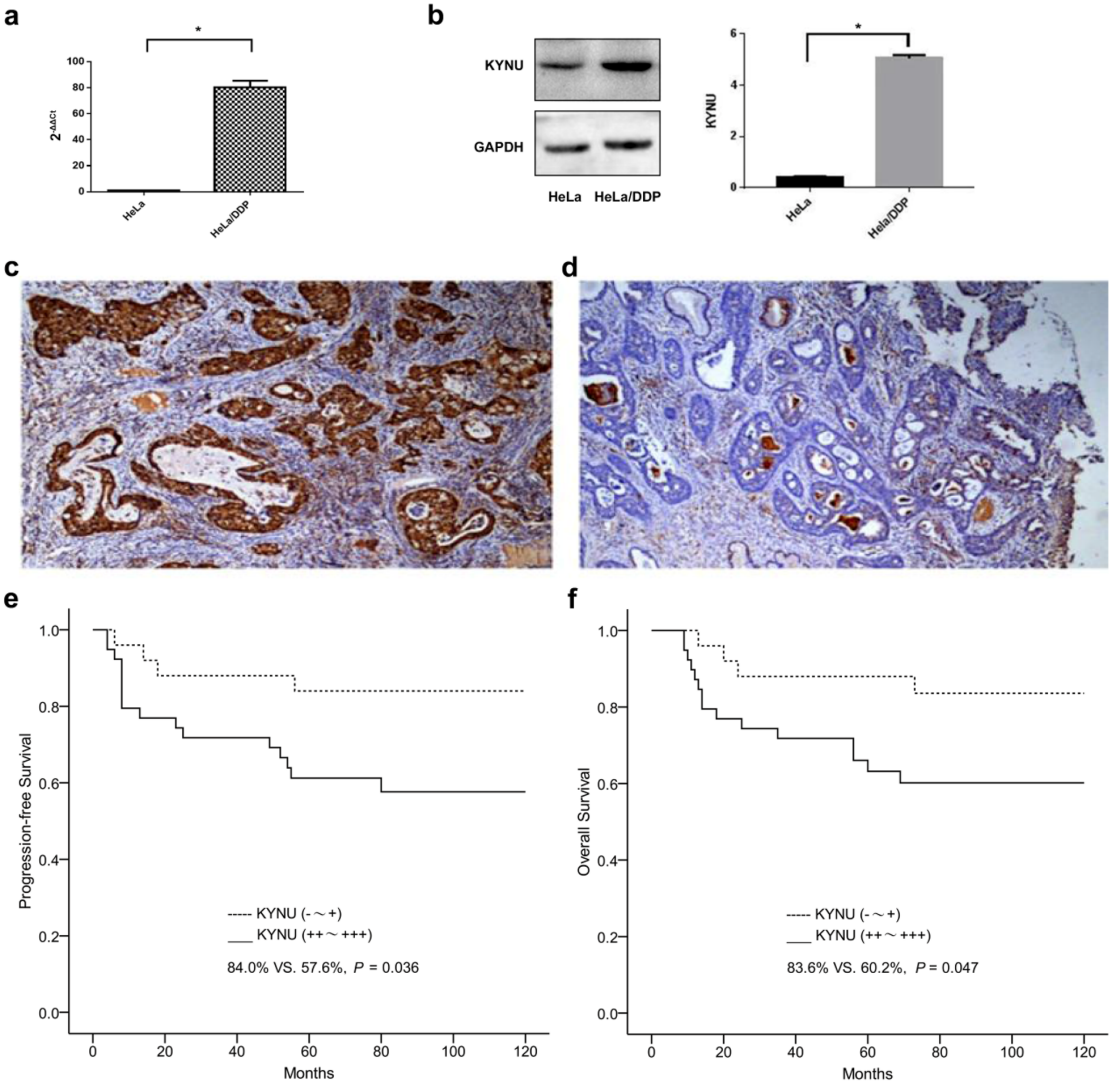
Increased KYNU expression in HeLa/DDP cells and tissues. **a** KYNU mRNA in the HeLa and HeLa/DDP cells. **b** KYNU protein in the HeLa and HeLa/DDP cells (the samples derive from the same experiment and that gels/blots were processed in parallel). **c** KYNU protein in the DDP-resistant tissues (+ + +). **d** KYNU protein in the DDP-sensitive tissues ( +). **e** Progression-free survival analysis of high and low KYNU expression. **f** Overall survival analysis of high and low KYNU expression. ^*^P < 0.05

The gene microarray (Affymetrix human transcript HTA2.0 mRNA) showed that the greatest difference between the HeLa and HeLa/DDP cells was in the expression of KYNU mRNA (*P* < 0.05, Table 1). Compared to the HeLa cells, KYNU mRNA expression was high in the HeLa/DDP cells (*P* < 0.05, Fig. 1a). The Western blot results revealed the over-expression of KYNU protein in the HeLa/DDP cells compared to the HeLa cells (Fig. 1b). Further, the expression of KYNU was slightly higher in the DDP-resistant ADC tissues than in the DDP-sensitive ADC tissues (*P* > 0.05, Table 2, Fig. 1c–d), and the Kaplan–Meier survival analysis indicated that ADC patients with higher KYNU expre

**Table 1:** The top five differentially expressed genes of HeLa and HeLa/DDP in the gene microarray

	HeLa vs. HeLa/DDP	Fold Change	p-value
KYNU	HeLa down	− 28.82	0.00000023
CASP1	HeLa down	− 18.03	0.00000074
C4BPA	HeLa down	− 16.00	0.00000044
HGD	HeLa high	13.38	0.00001220
FLJ22447	HeLa down	− 11.96	0.00000596

ssion levels had worse prognoses (Fig. 1e–f).

**Table 2:** The expression levels of KYNU protein in DDP-resistant and DDP-sensitive ADC tissues

Group	n	KYNU		χ^2^	*P*
		− ~ +	+ + ~ + + +		
DDP-resistant	33	7	26	3.344	0.067
DDP-sensitive	20	9	11		

### KYNU mRNA knockdown influences the invasion, migration, proliferation, and apoptosis rate of HeLa/DDP cells in a cisplatin environment

**Fig. 2:**
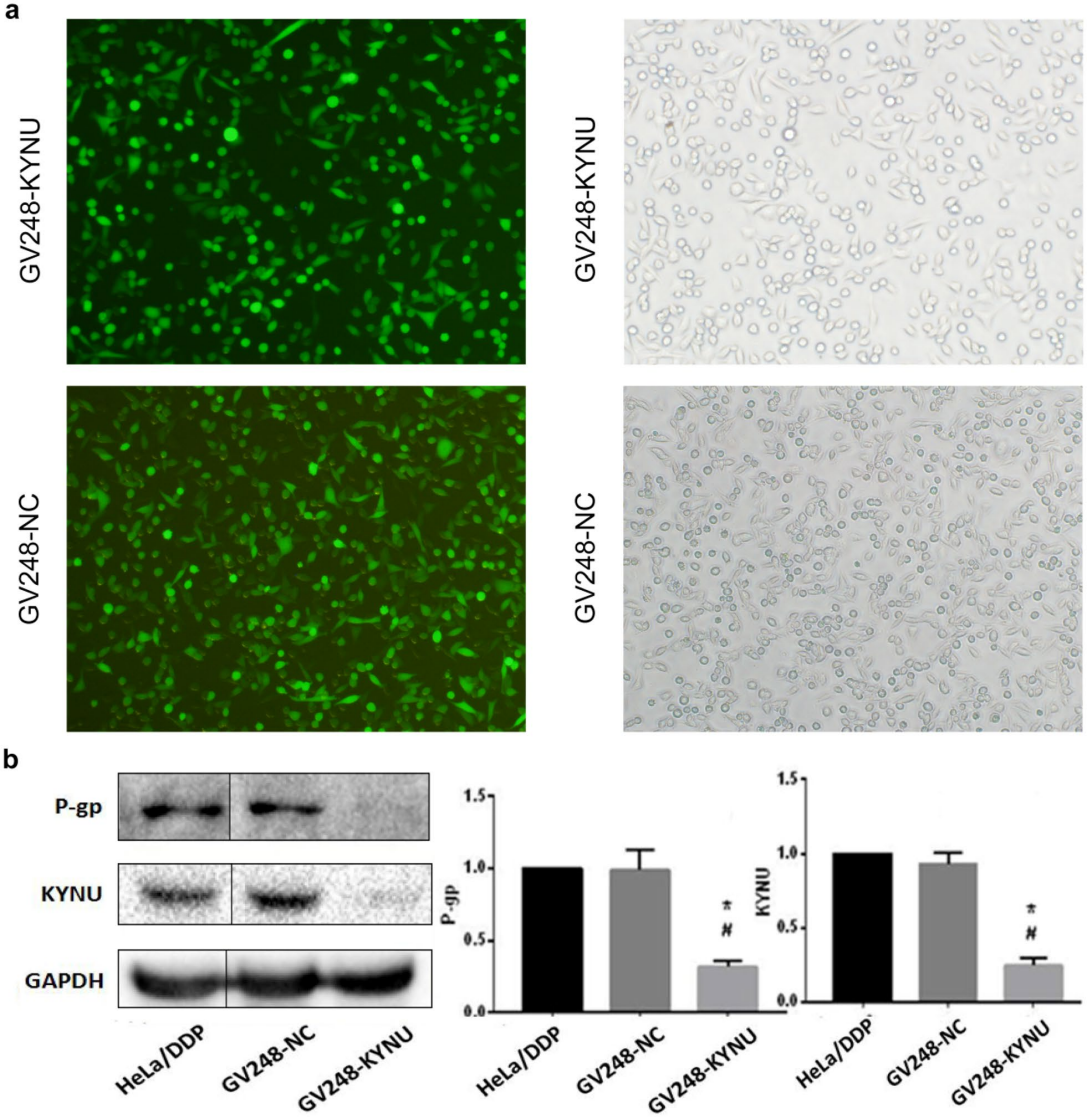
KYNU and P-gp expression (cropped gels, but the samples derive from the same experiment and that gels/blots were processed in parallel.) levels in the GV248-KYNU, GV248-NC, and HeLa/DDP cells. ^*^P < 0.05, when compared to HeLa/DDP, ^#^*P* < 0.05, when compared to GV248-NC

We used a lentivirus to create a Hela-DPP derivative cell line with shRNA-mediated stable KYNU mRNA knockdown. KYNU mRNA knockdown cell lines (GV248-KYNU) and negative control cell lines (GV248-NC) showed green fluorescence 24 h after lentivirus infection, and fluorescence expression was the strongest 72 h later. The transfection efficiency was 99% after screening with 2 μg/mL puromycin (Fig. 5). The expression levels of KYNU and P-gp protein were significantly lower in the GV248-KYNU group than in the HeLa/DDP and GV248-NC groups, and the differences were statistically significant (*P* < 0.05). There were no significant differences between the GV248-NC and HeLa/DDP groups with respect to the expression levels of KYNU and P-gp protein (*P* > 0.05, Fig. 2).

**Fig. 3:**
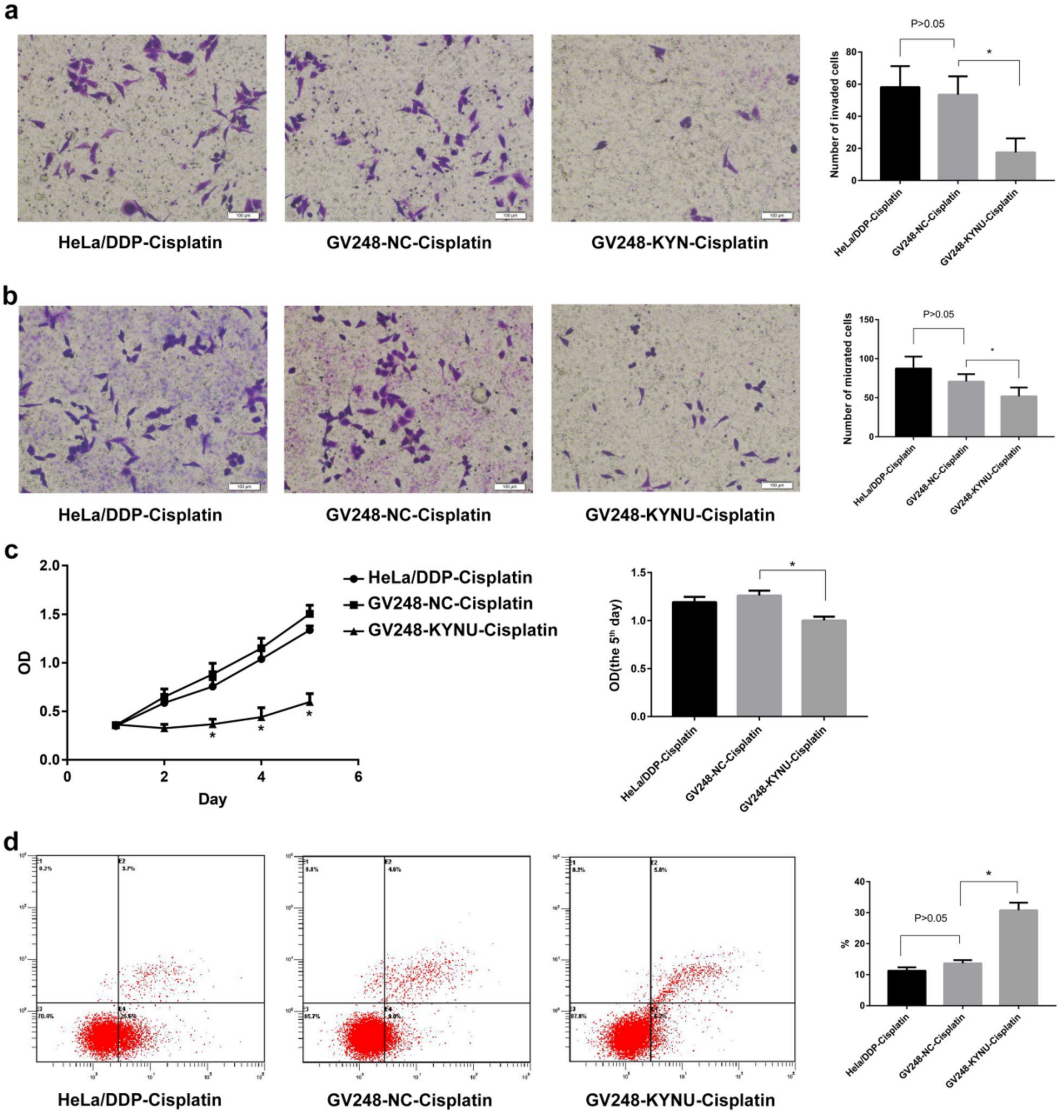
Effects of KYNU knockdown on HeLa/DDP cell proliferation (**a**), invasion (**b**), migration (**c**), and apoptosis (**d**)

After KYNU mRNA knockdown, the invasion, migration, and proliferation of HeLa/DDP cells in the cisplatin environment significantly reduced (*P* < 0.05), and their apoptosis rate significantly increased (*P* < 0.05, Fig. 3).

### KYNU knockdown improved the DDP sensitivity of ADC in BALB/c mice injected with GV248-KYNU cells

#### Effect of KYNU knockdown on DDP sensitivity of ADC in BALB/c mice injected with GV248-KYNU

**Fig. 4:**
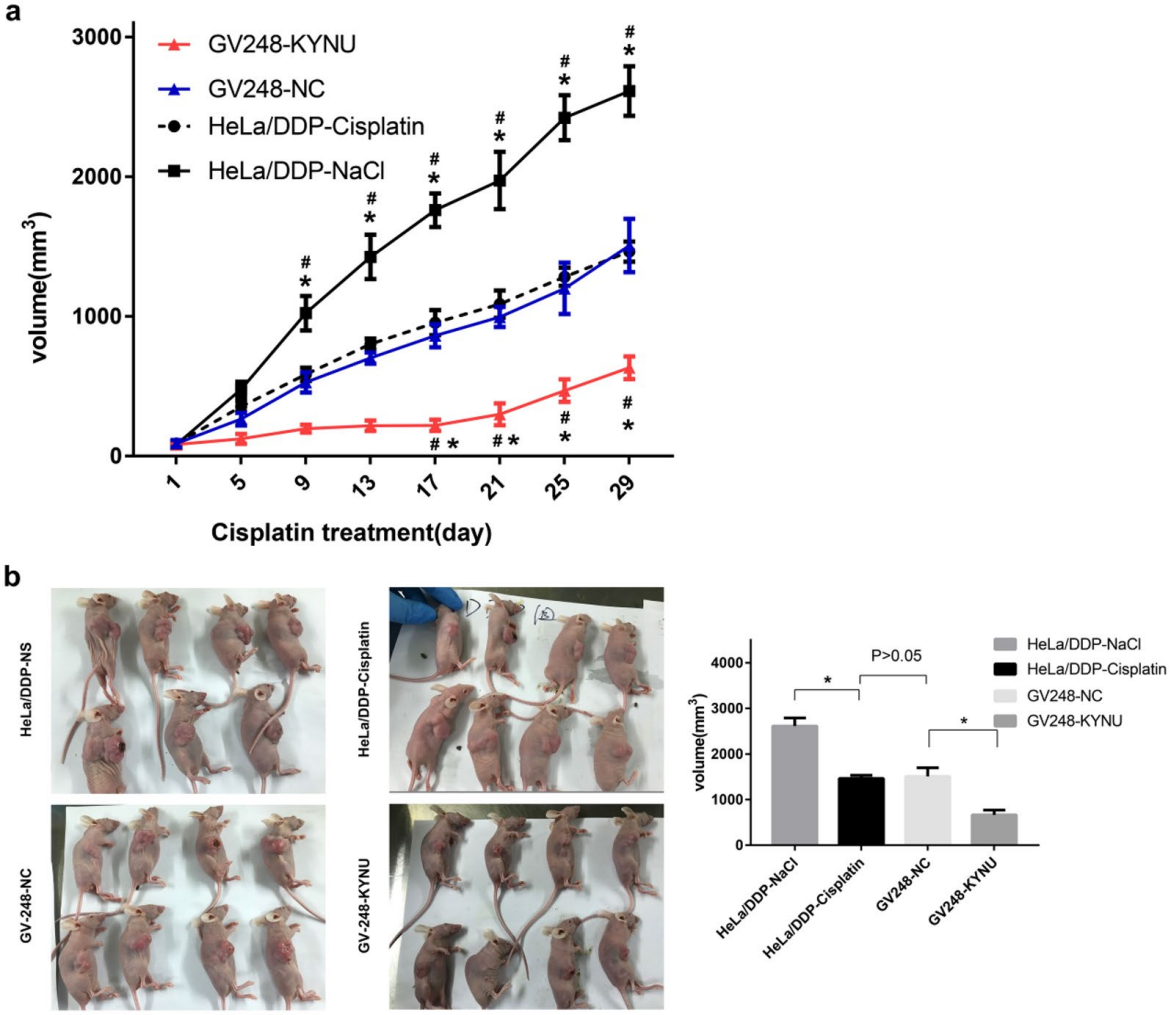
Effects of KYNU mRNA knockdown on the growth of mice. **a** The volume of transplanted tumors at different time points. **b** The volume of transplanted tumors on the 29th day after chemotherapy. ^*^P < 0.05, when compared to HeLa/DDP, ^#^*P* < 0.05, when compared to GV248-NC

A mouse model of subcutaneous transplanted tumors was successfully constructed in this study. When the transplanted tumors grew to about 100 mm^3^, cisplatin chemotherapy was intraperitoneally performed at a dose of 3 mg/Kg every other day for a total of seven days. The mice in the HeLa/DDP-NS group, which were intraperitoneally injected with normal saline, showed the fastest tumor growth rate and the largest tumor volume. The mice in the GV248-KYNU-cisplatin group had the slowest tumor growth rate and the smallest tumor size, and the differences were statistically significant compared to the HeLa/DDP and GV248-NC groups (*P* < 0.05). Notably, there were no significant differences between the GV248-NC-cisplatin and HeLa/DDP-cisplatin groups in terms of tumor growth rate and volume (*P* > 0.05) (Table 3, Fig. 4  ).

**Table 3:** Volume of transplanted tumors at different time points (mm^3^)

Day after chemotherapy	GV248-KYNU	GV248-NC	HeLa/DDP-Cisplatin	HeLa/DDP-NS
1	82.6 ± 15.1	89.1 ± 62.8	89.2 ± 15.7	82.1 ± 16.2
5	123.2 ± 85.2	265.1 ± 110.4	354.8 ± 95.8	478.7 ± 129.0
9	196.7 ± 66.9	527.7 ± 176.5	585.3 ± 117.5	1023.4 ± 302.3
13	217.9 ± 92.2	701.7 ± 98.0	802.2 ± 93.4	1425.5 ± 388.0
17	219.9 ± 98.2	861.2 ± 203.5	955.1 ± 219.1	1760.2 ± 293.5
21	298.5 ± 191.8	995.3 ± 175.9	1087.3 ± 239.4	1972.5 ± 503.5
25	468.6 ± 199.4	1200.0 ± 450.2	1283.6 ± 156.6	2421.6 ± 395.2
29	632.5 ± 198.0	1507.2 ± 468.4	1463.1 ± 175.4	2612.4 ± 436.5

#### Effects of KYNU mRNA knockdown on the survival of mice after chemotherapy

**Fig. 5:**
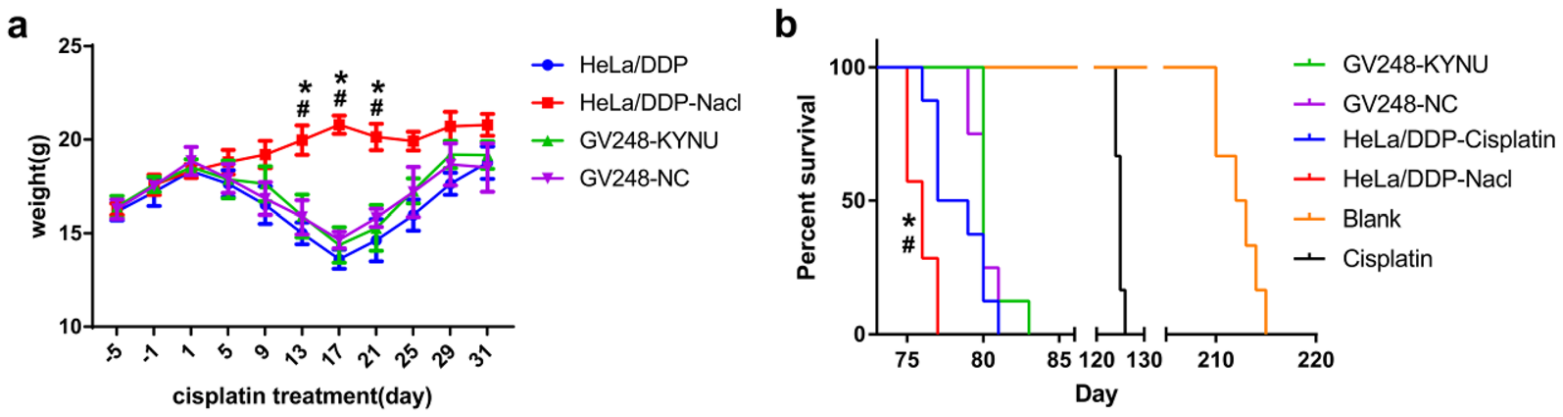
Effects of KYNU mRNA knockdown on the weight **a** and survival **b** of mice after chemotherapy. Blank: Mice without any interference. Cisplatin: Mice that underwent chemotherapy but without HeLa/DDP cell transplantation. ^*^P < 0.05, when compared to HeLa/DDP, ^#^*P* < 0.05, when compared to GV248-NC

The natural mean survival period of the mice was 210 days, and the mean survival of the mice that underwent chemotherapy without HeLa/DDP cell transplantation was 124 days. The mean survival periods of the mice in the HeLa/DDP-NS, HeLa/DDP-cisplatin, GV248-NC, and GV248-KYNU groups were 75, 76, 79, and 80 days, respectively. There were no significant differences in body weight or overall survival time between the HeLa/DDP-cisplatin, GV248-KYNU-ciplatin, and GV248-NC-cisplatin groups (Fig. 5).

#### Effects of KYNU mRNA knockdown on the morphology of HE staining in tumor-bearing mice after cisplatin chemotherapy

**Fig. 6:**
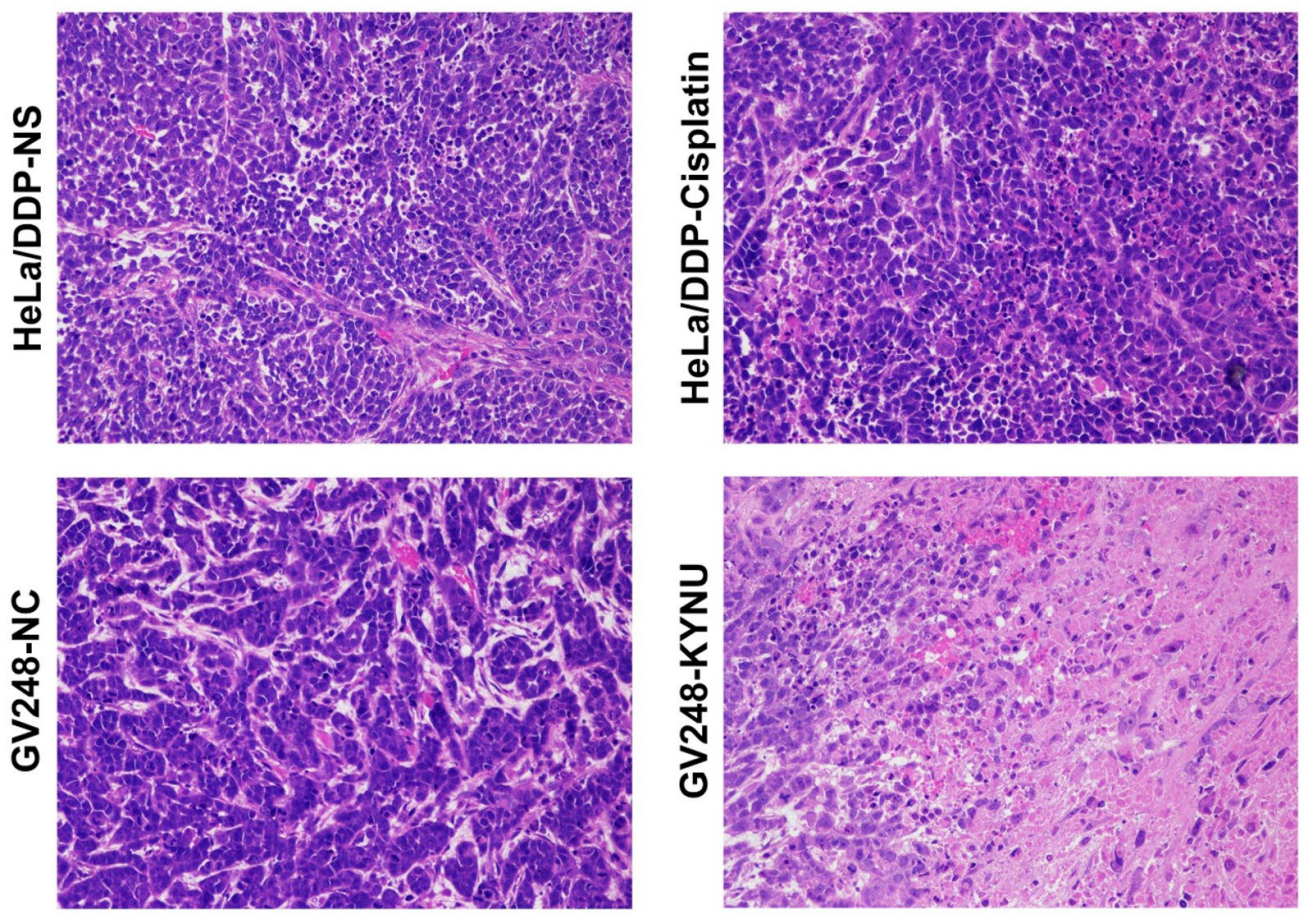
HE staining of the transplanted tumors of mice in each group

The mice were dissected to observe whether there was any tumor metastasis. The tumor specimens were removed for paraffin sectioning, and pathological changes in the transplanted tumors were observed via HE staining. No tumor metastasis was observed in any of the groups. The mice in the GV248-KYNU-cisplatin group showed lighter nuclear staining, smaller tumor nests, less atypia, and more nuclear mitosis, flake coagulation, and focal necrotic substances than the mice in the other three groups. However, the HeLa/DDP-NS, HeLa/DDP-cisplatin, and GV248-NC groups had more tumor cells, vigorous growth, and nuclear mitosis and more abundant cancer nests than the GV248-KYNU-cisplatin group (Fig. 6).

#### Effect of KYNU knockdown on the expression of P-gp, MRP1, GST-π, Bax, Bcl-2, and CD34 protein

**Fig. 7:**
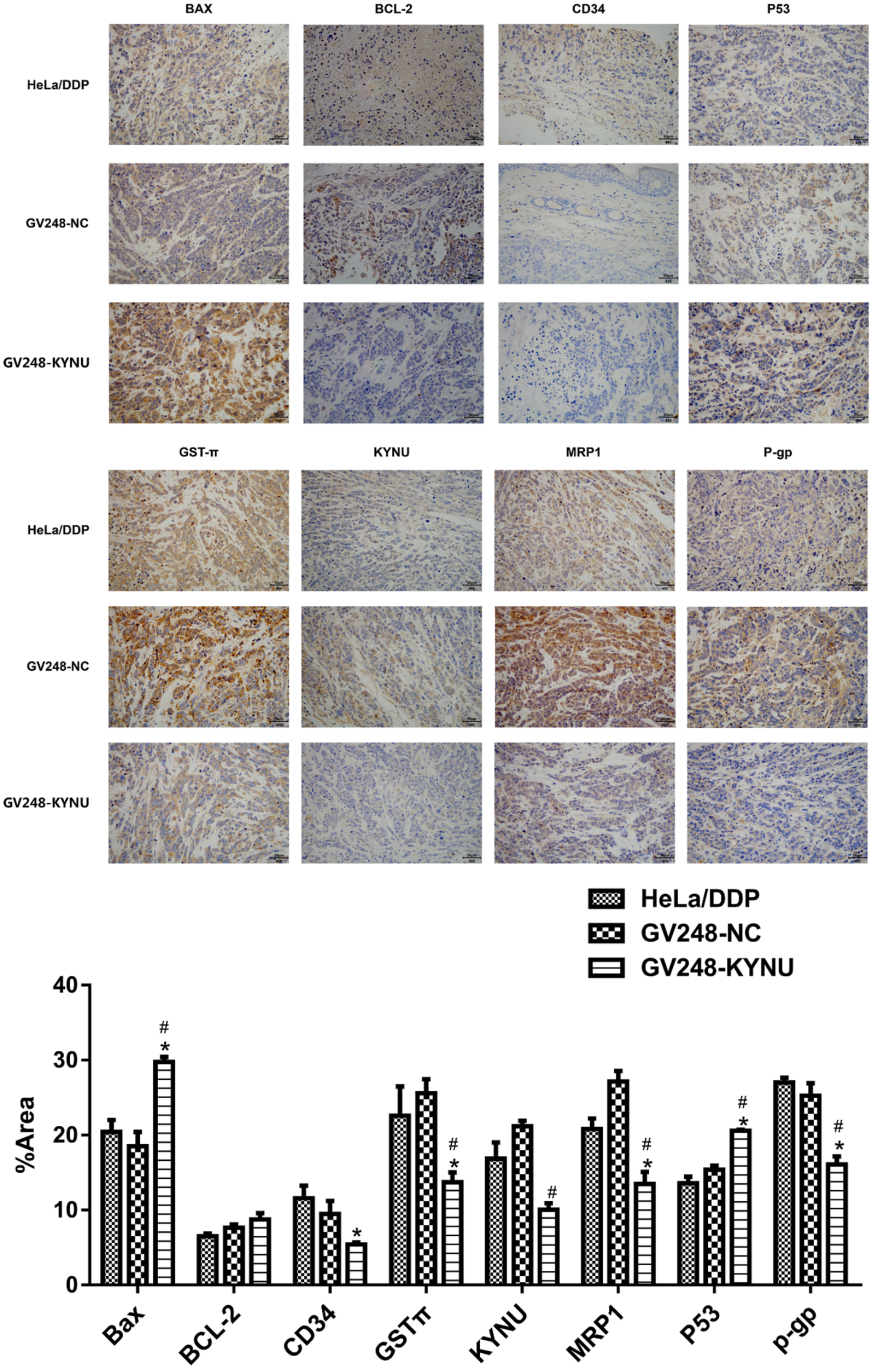
The expression levels of KYNU, P-gp, MRP1, GST-π, Bax, Bcl-2, and CD34 proteins in tumor-bearing mouse tissues. ^*^P < 0.05, when compared to HeLa/DDP, ^#^*P* < 0.05, when compared to GV248-NC

After KYNU mRNA knockdown, KYNU, CD34, and the drug-resistance related proteins P-gp, MRP1, and GST-π in the GV248-KYNU-cisplatin group were significantly downregulated compared to the HeLa/DDP-cisplatin and GV248-NC-cisplatin groups (*P* < 0.05). The expression level of the proapoptotic regulatory protein Bax was significantly higher in the GV248-KYNU-cisplatin group than in the HeLa/DDP-cisplatin and GV248-NC-cisplatin groups (*P* < 0.05). The antiapoptotic protein Bcl-2 was upregulated a little more in the GV248-KYNU-cisplatin group than in the other two groups, but the difference was not statistically significant (Fig. 7).

## Discussion

Chemoresistance is a leading cause of chemotherapy failure in cervical ADC, but currently there is a lack of effective treatment options. Abnormal metabolism is one of the basic characteristics of a tumor. Changes in metabolic pathways and metabolites regulate the life activities of tumor cells through mechanisms such as transcription, translation, posttranslational modification, and microenvironment changes, which, in turn, play crucial roles in inflammation, neurogenic diseases, tumor development, and drug resistance [8, 9]. KYNU is a key enzyme in the tryptophan pathway, through which more than 90% of human tryptophan is metabolized [10]. In recent years, the tryptophan–kynurenine pathway has received a lot of attention due to the immunosuppressive and cancer-promoting effects of its key enzymes and metabolites [11, 12]. In addition, a large number of studies have shown that KYNU plays an important role in carcinogenesis, tumor progression, and chemoresistance.

Gao et al. [13] reported that KYNU expression is closely related to the prognosis of pediatric acute lymphoblastic leukemia. The higher the expression, the worse the prognosis, indicating that KYNU is a carcinogenic factor in pediatric acute lymphoblastic leukemia. Ci et al. [14] found that a knockdown of KYNU expression could inhibit the proliferation of cutaneous squamous cell carcinoma by inhibiting the PI3K/AKT signaling pathway. Moreover, Li et al. [7] used gene chips to detect differentially expressed genes in drug-resistant and drug-sensitive samples of laryngeal cancer. Their results showed that KYNU was significantly upregulated in the chemotherapy-resistant group, and a receiver operating characteristic curve (ROC) analysis showed that KYNU was a good chemotherapy sensitivity index. In the present study, KYNU was overexpressed in HeLa/DDP cells and tissues and was associated with the poor prognoses of patients with ADC. After KYNU knockdown, the cisplatin sensitivity of nude mice with transplanted tumors increased, and the tumor growth rate slowed down. This indicates that KYNU is involved in the chemotherapy resistance of cervical ADC. Our results are consistent with the experimental results of the studies mentioned above. Notably, Liu et al. [15] found lower KYNU expression in breast cancer tissues than in lymphoid tissues and benign breast disease tissues. Furthermore, KYNU expression in breast cancer tissues was positively correlated with estrogen and progesterone receptor and negatively correlated with HER2 and Ki-67 expression, tumor size, and clinical stage. In addition, MTT assay results showed that highly expressed KYNU could inhibit the proliferation and migration of breast cancer cells, suggesting that KYNU is a tumor suppressor gene in breast cancer; this may be related to differences in tumor species and pathological patterns.

The proapoptotic regulatory protein Bax and the antiapoptotic regulatory protein Bcl-2 play important roles in cell apoptosis and drug resistance. In ovarian cancer cells, the downregulation of Bax and upregulation of Bcl-2 are associated with cisplatin resistance [16]. Bax mRNA has been found to be significantly downregulated in fluorouracilresistant colorectal cancer cells compared to wild-type HT-29 cells, suggesting that Bax is closely related to 5-FU resistance in colorectal cancer [17]. Decreased Bax/Bcl-2 ratio and caspase activity are the main mechanisms that cause the malignant glioblastoma cell line U87MG to be resistant to temozolomide (TMZ) [18] and the breast cancer cell line MCF-7 to be resistant to paclitaxel [19]. Bax mutations can also cause multidrug resistance [20]. In our study, the expression of the proapoptotic regulatory protein Bax was significantly upregulated after KYNU mRNA knockdown, the expression of the antiapoptotic protein Bcl-2 was slightly downregulated, and the Bax/Bcl-2 ratio increased. Furthermore, the expressions of the drug resistance-related proteins P-gp, MRP1, and GST-π were significantly downregulated, indicating that KYNU contributes to chemoresistance and the malignant progression of cervical ADC by regulating the expression of Bax.

In conclusion, KYNU deficiency could enhance DDP sensitivity by suppressing cell proliferation, migration, and invasion and promoting apoptosis in DDP-resistant ADC cells in vitro. KYNU knockdown improved the drug sensitivity of ADC samples in vivo. Thus, KYNU is involved in the chemotherapy resistance of cervical adenocarcinoma.

## Data Availability

All data generated or analyzed during this study are included in this article. Further enquiries can be directed to the corresponding author.
